# Sachem: a chemical cartridge for high-performance substructure search

**DOI:** 10.1186/s13321-018-0282-y

**Published:** 2018-05-23

**Authors:** Miroslav Kratochvíl, Jiří Vondrášek, Jakub Galgonek

**Affiliations:** 1 Institute of Organic Chemistry and Biochemistry of the CAS, Flemingovo náměstí 2, Prague 6, 166 10 Czech Republic; 2 Department of Software Engineering, Faculty of Mathematics and Physics, Charles University, Malostranské náměstí 25, Prague 1, 118 00 Czech Republic

**Keywords:** Substructure search, Small molecule databases, Molecule cartridges, Inverted indices

## Abstract

**Background:**

Structure search is one of the valuable capabilities of small-molecule databases. Fingerprint-based screening methods are usually employed to enhance the search performance by reducing the number of calls to the verification procedure. In substructure search, fingerprints are designed to capture important structural aspects of the molecule to aid the decision about whether the molecule contains a given substructure. Currently available cartridges typically provide acceptable search performance for processing user queries, but do not scale satisfactorily with dataset size.

**Results:**

We present Sachem, a new open-source chemical cartridge that implements two substructure search methods: The first is a performance-oriented reimplementation of substructure indexing based on the OrChem fingerprint, and the second is a novel method that employs newly designed fingerprints stored in inverted indices. We assessed the performance of both methods on small, medium, and large datasets containing 1, 10, and 94 million compounds, respectively. Comparison of Sachem with other freely available cartridges revealed improvements in overall performance, scaling potential and screen-out efficiency.

**Conclusions:**

The Sachem cartridge allows efficient substructure searches in databases of all sizes. The sublinear performance scaling of the second method and the ability to efficiently query large amounts of pre-extracted information may together open the door to new applications for substructure searches.

## Background

Compound retrieval is one of the most common purposes of chemical database cartridges. The ability of database cartridges to process queries accurately and efficiently has helped them become powerful tools to perform functions such as virtual screening, novelty checks, and compound activity predictions [1, 2]. Substructure search, in which the user inputs a part of a molecular structure and receives a set of all molecules that contain the given fragment as a substructure, can be valuable for searching through chemical databases.

The time required to deliver a substructure search result depends on the speed of the substructure matching algorithm that determines whether the given molecular fragment is a subgraph of a compound, and on the ability of the cartridge to quickly screen out compounds that can be identified as unable to match the query, so that they are not unnecessarily fetched from storage and verified by costly substructure matching. Because subgraph matching is an instance of a relatively hard subgraph isomorphism problem that is inherently exponential, improvements in screening are usually the major factor in expediting the search procedure. Screening is typically guided by substructural fingerprints that are stored in database indices for rapid processing [3].

Here, we present Sachem, a new open-source chemical cartridge that aims to run substructure search queries on the largest publicly available datasets (specifically on more than 90 million compounds in the PubChem database) in an insignificant amount of time. In addition, the performance of the cartridge is potentially scalable to much larger database sizes. Although the scalability of cartridges is a crucial concern for long-term deployment^1^, it is seldom discussed in the literature, and not sufficiently assured by available open-source software.

Development of Sachem was motivated by two major factors: First, substructure search is sometimes used as a frequently-called subroutine of other algorithms or analyses (e. g., complex virtual screening schemes [4] or novel interfaces for substructure search [2]); improvements of its performance are therefore directly reflected in the performance of the entire algorithms. Second, researchers usually benefit from the ability to interactively run and refine substructure queries using a platform such as a web service to quickly access available information about the compounds; the performance and efficiency of the cartridge implementation directly lower the running cost of such a web service.

### Fingerprint-based screening

The typical abstraction that guides the screening phase of the search is substructural fingerprints [3, 5, 6]. These differ from other forms of fingerprints (e.g. similarity fingerprints) by specifying an extra requirement: a feature present in a structure must also be present in all its structural extensions (i.e. the set of fingerprints with a sub-fingerprint relation must form a partially ordered set that is homomorphic to the set of chemical structures partially ordered by subgraph relation). Further we discuss only the substructural fingerprints.

The fingerprints used in currently available cartridges were either designed manually or generated by automatically identifying and collecting features from molecule graphs. Manual design of fingerprints usually results in a compact feature set that captures the available domain-specific knowledge of search-related substructures; these include the substructural fingerprints from the CDK library, OrChem fingerprints, CACTVS and MACCS descriptors [7–10], and many others [6]. Fixed fingerprint content is not future-proof—new features or statistical changes in the data require the fingerprint to be re-designed. This drives the need to generate fingerprints automatically, using only general assumptions. Typical auto-generated fingerprints include descriptors based on atom-count, atom-pairs, atom-paths, sub-graphs, wildcard patterns, and ring-connectivity [11, 12].

Technically, fingerprints are representable either as bit vectors, in which each bit marks the presence of a feature; or numeric vectors that also encode repetitions of each feature, which can also be encoded as bit vectors [13]. The optimal method of storage and indexing depends on the fingerprint properties. Vectors of hand-designed fingerprints (typically identifying at most thousands of features) can be stored as simple relational-database columns and indexed by B-trees [8], stored as arrays and indexed by generalized inverted search-tree indices [14], or stored as inverted feature vectors or bitmap indices to allow rapid processing [15].

Auto-generated fingerprints typically produce vectors with large numbers of descriptors: for example, in the ChEBI compound database [16] (which currently contains cca. $10^5$ compounds), we can identify hundreds of different atom variants (atomic numbers, isotopes, and charges), approximately $10^3$ different ring structures, $8\cdot 10^5$ different radial structures^2^ of radius 4, and $1.5\cdot 10^6$ different graph substructures with a maximum of 6 bonds. Cartridges usually exploit the sparsity of the resulting vectors to store them efficiently and represent each vector as a list of feature indices, or more generally, index-count pairs.

There are two common approaches for storing sparse fingerprints. The indices may be folded down by hashing feature indices to smaller integers, possibly using techniques similar to Bloom filters [18, sec. 5.3]. The resulting reduction in dimensionality makes it possible to store the folded vectors using the same methods as for short feature vectors [6]. Alternatively, the unfolded feature lists can be stored in inverted indices as is, without information loss caused by possible hash collisions in folding [13, discussion on p.12]. Indexing methods available in text-search databases (e. g. Apache Lucene, Lucy, Solr [19–21], and ElasticSearch [22]) scale well to the required count of distinct features and molecules. Inverted indices usually exploit statistical properties of the data to reduce the consumption of storage: posting lists of text-search databases typically contain small delta-encoded integers; similar encodings have been used for chemical databases [23].

Sometimes a mixed approach for reducing the dimensionality of the fingerprints is taken. Machine learning methods can be used to simulate expert decisions about the viability of individual fingerprint keys, and the resulting fingerprint may be as good and compact as a hand-designed one [24–26].

### Available open-source cartridges

There are several available open-source cartridges that support molecule storage in a database retrieval using a substructure search query. These currently include OrChem [8], RDKit [27], Bingo [15], pgchem [14] and Mychem [28].

*OrChem* uses Oracle as the back-end database. Its 789-bit fingerprint contains hashes of three-atom SMILES substructures in the first 95 bits; the rest of the bits is specified manually, in many cases using structural patterns. OrChem also stores 15 integer values for each molecule that describe counts of specific atoms and bond types. Each fingerprint bit and integer value is stored in a separate table column, and these columns are then indexed using a B-tree index.

*RDKit* provides a cartridge functionality with the PostgreSQL database. Fingerprints used for screening are pattern fingerprints, which hash several common structural features defined by SMARTS patterns. The size of the fingerprint can be customized, by default the hashes are folded to a 2048-bit array. Substructural fingerprints are indexed in a custom GiST index.

*Bingo* uses a 2584-bit substructural fingerprint based on hashed cyclic subgraphs (up to 8 atoms) and hashed sub-trees (up to 7 atoms). A small part of the fingerprint is reserved to count atom types, charges, and isotopes. Bingo can use Oracle, PostgreSQL, or SQL Server as a back-end. Fingerprints are indexed using a custom index type.

*pgchem* uses slightly modified FP2 and FP3 fingerprints from OpenBabel [29] concatenated to one 1536-bit fingerprint. FP2 is a path fingerprint that indexes linear subgraphs with a maximal length of 7 atoms, FP3 is based on manually defined SMARTS patterns. Fingerprints are stored as a custom data type with a GiST index.

*Mychem* is a chemical cartridge for MySQL. It allows computation of FP2, FP3, and FP4 fingerprints from OpenBabel. Mychem does not provide any direct way to index the fingerprints to allow substructure screening.

## Implementation

In this paper, we describe the implementation of *Sachem*, a new cheminformatic cartridge. Two different fingerprints and corresponding indexing and searching methods implemented in Sachem can be used.

The first method, called *Sachem/OrChem*, is a major performance-oriented modification of the substructure-search part of the OrChem database cartridge [8]. The second method, *Sachem/Lucy*, is an extension of the previous, designed to maximize the search screen-out rate by indexing a large number of automatically specified substructural fingerprint bits in a text-search database.

### Subgraph matching

Sachem was originally intended as a port of OrChem to the PostgreSQL database, which was motivated by licensing considerations. The subgraph matching functionality is therefore based on the original OrChem approach. However, we made multiple optimizations, which led to a complete rewrite of the matching part.

The text-based molecule storage format of OrChem was replaced with a compact binary format that allows faster fetching and processing of the molecular data in the substructure matching algorithm (many other cartridges take a similar approach for preparing the data). The compacted molecules are automatically stored in a static memory-mapped index file, so that the required data can be accessed without substantial overhead. The VF2 algorithm implementation was ported to C language and optimized for performance, resulting in a total speedup of more than $10^3$. Two major speed-ups were achieved by avoiding all memory allocations in the performance-critical part of substructure matching, and by precomputing and caching all data required by the matching algorithm. In the original OrChem, some frequently accessed properties of atoms and molecules, such as implicit hydrogen counts, were unnecessarily recomputed several times during each substructure match.

### Fingerprint structure

Sachem/OrChem uses the fingerprints that were defined in the original OrChem but modified to support CDK 2.0 and use the aromaticity detection algorithm that was newly added to CDK to allow contributions from exocyclic $\pi$-bonds.

The fingerprints used in Sachem/Lucy are defined as follows:

- The occurrence of each distinct atom type found in a compound is considered as a distinct fingerprint bit.
- Using the SSSR algorithm [30], we retrieved all smallest rings of all compounds in the ChEBI [16] database. Each distinct discovered ring is considered a substructural pattern for a fingerprint bit.
- All connected subgraphs of non-hydrogen atoms with a maximum of one ring and a limited number of bonds were also considered a fingerprint bit. The bond limit is parameterized as the GraphSize parameter, set to 7 by default. The single ring condition was selected to efficiently solve canonization problems with multi-ring structures. For the fingerprint, the subgraph is converted to a number using a tree-hashing algorithm that consecutively removes leaves until only the ring remains; the ring is then hashed in its lexically minimal rotation.
- Multiplicity of features was encoded by creating a new fingerprint bit for each power of 2 of the repetitions. Other systems (including OrChem [8] and RDKit [27]) use a similar encoding of multiplicity.

The resulting fingerprints are suitable for storage in text-search databases. In the PubChem database, the Sachem/Lucy method identified 18.7 million distinct feature descriptors with approximately 860 identified features per compound on average, which is comparable to natural-language data at which text-search databases are targeted.^3^

### Storage, indexing, and processing details

In addition to using a different database back-end and matching algorithm, Sachem/OrChem differs from the original OrChem in the indexing method used. Instead of B-tree based indices, Sachem/OrChem uses a memory-mapped inverted bitmap index to speed up searching in fixed-size fingerprints.

Sachem/Lucy uses fingerprints as defined in "Fingerprint structure" section instead of OrChem fingerprints, and indexes them in the Apache Lucy database [20] (hence the name). Apache Lucy is a port of the well-established Apache Lucene [19] text-search database to C. Our choice of Apache Lucy was motivated by the relative efficiency of C implementation compared to alternatives.

**Fig. 1:**
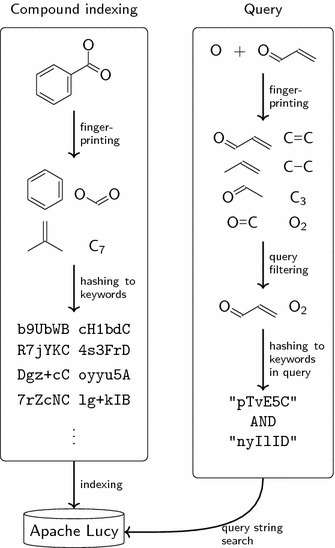
Fingerprint processing in Sachem/Lucy. The left box shows a molecule from the compound database being broken into distinct substructural features by fingerprinting (only 4 are shown for demonstration); these are converted to keyword-like descriptors by hashing and stored in Apache Lucy. The right box shows the querying process. The query is broken into substructural features. These are then filtered to only include features with reasonable filtering power; the result is converted to keyword descriptors to build a text query, which is in turn run on Apache Lucy

The fingerprints were adapted for storage in Apache Lucy as follows: identification of each fingerprint bit is encoded to a 36-bit integer and converted into a corresponding 6-byte base64 word suitable for being stored as a term in text documents. The resulting keywords are concatenated to a space-separated string and indexed as documents using a simple whitespace analyzer. The overall process is shown in Fig. 1.

From the user’s point of view, this adaptation to external indexing is called transparently from the PostgreSQL interface, which is common to both implementations.

### Screening performance optimization by bit selection

In text-search databases, the presence of a keyword in the query forces the database to traverse the corresponding part of the inverted index, which is particularly costly for long lists associated with frequently occurring keywords. This introduces a tradeoff—in our setting, specifying a more precise query by including more fingerprint bits may improve the screen-out rate, but at the cost of increased overhead to process more data.

To balance the factors in this tradeoff and accelerate the querying process, our method simply discards the bits from the query fingerprint that are not significant in terms of filtering power. Using the resulting *filtered query* dramatically reduces the overhead needed to traverse the indices, and causes only a small increase in false positives. The query fingerprint bit-reduction algorithm performs the discarding in two steps: 1) it uses the information about subsumption to discard redundant bits and 2) it decides whether to discard less relevant bits based on their statistical relevance for search.

The first step of discarding is implemented in the query fingerprinting procedure, and is performed separately for all fingerprint types and repetition encoding. The procedures discard fingerprint bits if their presence is directly subsumed by others. This step does not lead to any additional false positives.

**Fig. 2:**
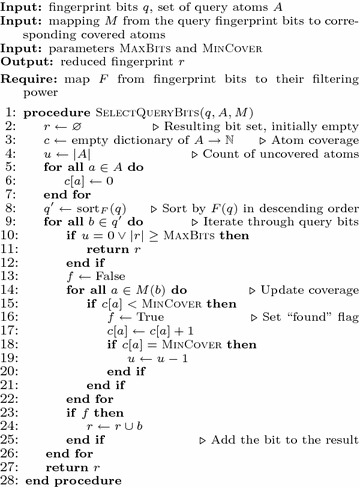
Algorithm to select fingerprint bits most relevant to the given query. Upon input, it receives set *q* of fingerprint bits from the first step of the fingerprint reduction algorithm, set *A* of atoms that are in the query, and mapping *M* from the query fingerprint bits to corresponding covered atoms. The algorithm is parameterized by the positive integers MaxBits and MinCover. The MaxBits parameter is a hard limit on the count of bits in the reduced fingerprint, and the MinCover parameter sets the minimal count of distinct fingerprint bits in the reduced fingerprint that cover each atom present in the query molecule. The algorithm assigns a covering counter (initially set to zero) to each atom of the query molecule. The query fingerprint bits are then traversed in descending order of filtering power. For each bit, it is determined whether there exists a query atom that is covered by the bit information. If its associated counter is less than MinCover, all counters of atoms covered by the bit are increased, and the bit is added to the resulting reduced query fingerprint; otherwise, the bit is discarded. During the development, we experimentally determined that 2 and 32 are suitable values for MinCover and MaxBits, respectively. The filtering power of distinct bits (function *F*) is obtained by counting the relative occurrences of the bits in the dataset. The resulting *F* is portable to other datasets; re-computation is needed only after substantial statistical changes in data

The second step attempts to discard bits that are expected not to contribute significantly to screen-out. The decision about discarding individual bits uses a pre-computed table with the relative filtering power of each distinct bit and extra information that connects query bits to corresponding “covered” query atoms. We considered an atom to be covered by a fingerprint bit if it is contained in part of the molecule that has caused the bit to be non-zero. From this information, the algorithm finds a small set of fingerprint bits that is expected to cover the query well and have sufficiently high filtering power to keep the resulting decrease in screen-out ratio relatively low. The *bit-selection algorithm* for the second step of fingerprint bit-reduction is detailed in Fig. 2.

## Results and discussion

We performed a comprehensive benchmark to assess the performance and scaling advantages of Sachem and compare it with other available cartridges.

### Benchmark setup

We ran the same benchmark—storing a dataset in a cartridge and running a set of substructure queries on it—on all combinations of cartridges and datasets.

We recorded the time needed for overall query processing and for the screening phase. We counted the compounds that passed the screening and verification phases to compute screening precision and selectivity.

#### Benchmarked cartridges

We benchmarked three variants of Sachem: the two versions described in this work, Sachem/OrChem and Sachem/Lucy, as well as *Sachem/eCDK*, which is a modification of Sachem/OrChem that differs only in fingerprinting procedure—it uses the ExtendedFingerprinter from the CDK library instead of OrChem fingerprints.

For our comparison, we focused on methods that are available in the form of cartridges with a SQL-based front-end. We included OrChem [8] (version 1.3.1), RDKit [27] (the PostgreSQL-based cartridge implementation with 2048-bit fingerprint, version 2017.09.1), Bingo [15] (the PostgreSQL-based cartridge variant, version 1.8.0-beta) and pgchem [14] (version 1.3-GiST).

All these cartridges use PostgreSQL, version 9.6, as a back-end database, except for OrChem, which we ran on Oracle database, version 12c.

Thorough comparisons of many other cartridges are available elsewhere [31–34], and these can be used to relate the performance of Sachem to cartridges not included in our benchmark.

#### Datasets

To assess the performance scaling behavior of the cartridges, we ran the benchmarks on three datasets of different size. The *94M* dataset consists of all compounds in the PubChem database snapshot from August 2017 (PubChem contained just under 94 million molecules at the time). We randomly selected 10 million compounds from the 94M dataset to form the *10M* dataset, which was further randomly reduced to 1 million compounds in the *1M* dataset.

We chose to select the 1M dataset as a subset of 10M to avoid the effect of outliers (described in more detail in "Performance outliers" section). Because some queries take much longer than average to be processed on certain molecules (e. g. cases in Fig. 4a, b), an unfortunate selection of molecules in the 1M dataset could make cartridges perform worse on it than on the 10M dataset. Selection as a subset ensures that the 10M dataset is at least as hard to process as the 1M dataset.

Some cartridges failed to index several molecules from the datasets; these errors are briefly summarized in Table 1.

**Table 1:** Overview of indexing and searching errors

Cartridge	Indexing failures			Rejected queries	
	1M	10M	94M	*n*	reason
Bingo	105	1024	9754	0	
OrChem	2	4	–	12	Unsupported aromatic bond in SMILES
pgchem	30	255	2527	146	Fragmented SMILES, queries with [*]
RDKit	72	707	6911	4	Chemical structure considered invalid
Sachem	0	0	0	0	

Measurements are slightly influenced by errors that some cartridges exhibited during benchmarking, due to both indexing and searching errors. Indexing errors are primarily reported as unacceptable data in the SDF files from PubChem, most frequently as invalid atom valences or stereochemistry. Note that Bingo beta version can lower the number of indexing errors by using algorithms that work with ‘incorrect’ structures (this feature is disabled by default)

#### Query set

Design of a good, unbiased query set for benchmarks is complicated. It is not possible to derive such a set from a pre-existing statistic of common user queries, which favors popular queries viable for recently conducted research, or from identified features in the database, which favors queries based on the feature identification method and produces a bias towards database content.

To more easily draw comparisons with our systems, we re-used queries that have been benchmarked by other researchers available in Substructure Query Collection (SQC) [35]. SQC includes queries used by Ehrlich and Matthias [36] for a systematic benchmark for substructure search algorithms, and user queries collected from live software testing.

The collected queries are slightly biased toward simpler ‘explorative’ queries, but still represent a valid sample of queries in a publicly accessible database.

Of the 3488 queries present in the SQC query set, we removed 159 that are not supported by some of the cartridges; details can be found in Table 1.

#### Benchmarking hardware

All benchmarks were performed on CentOS Linux 7.4 running on virtualized Intel Haswell CPUs clocked at 2.6 GHz with 512 GB RAM; benchmarked software parts were run single-threaded. Results from the first runs of the benchmarks were discarded to allow the programs to cache hot data.

**Fig. 3:**
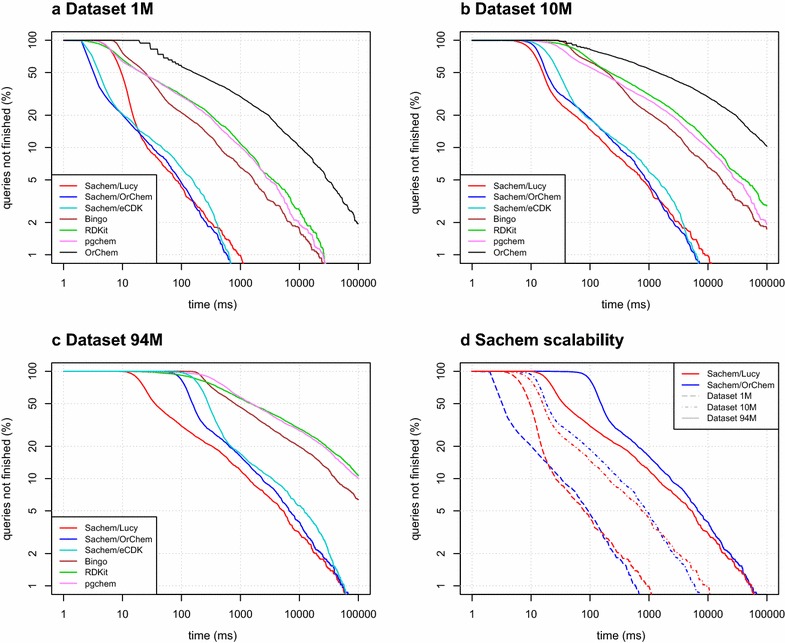
Overall performance comparison. Log-times in milliseconds are plotted on the *x* axis and log-percent of queries that took more than the plotted time are plotted on the *y* axis. Plots **a**–**c** show the performance comparison of Sachem/Lucy and Sachem/OrChem to other benchmarked cartridges on all datasets. The original OrChem was not benchmarked on the largest dataset due to the lengthy time that would be required to finish the benchmark. Plot **d** is a comparison of timings for Sachem/Lucy and Sachem/OrChem on all datasets that shows the scaling advantage of Sachem/Lucy

### Overall query performance

The overall timing results are summarized in plots in Fig. 3.

OrChem-based variants of Sachem outperformed all cartridges except Sachem/Lucy on all datasets. Sachem/OrChem was faster by an average factor $8\times$ than Bingo, which slightly outperformed the RDKit and pgchem cartridges. The ordering of cartridges by performance was often mixed—RDKit clearly performed better than Bingo on faster queries, but the performance advantage was lost on complicated queries.

Thanks to improvements in screening (measured separately as described in "Screening efficiency" section), Sachem/Lucy outperformed all other cartridges on the largest dataset by a wide margin. This advantage was partially lost on smaller datasets on fast queries, for which the Sachem/Lucy processing time was dominated by preparing the complicated fingerprint (less than 10 ms in most cases) and by increased overhead for the inverted index processing. However, as the performance disadvantage of Sachem/Lucy is at most approximately 10 ms for the majority of queries, we do not consider it to be a significant drawback. See Fig. 3d for a side-by-side comparison on all dataset sizes.

The same plot also illustrates the scalability improvement in Sachem/Lucy. While the time required by Sachem/OrChem to answer all queries was roughly linear with increasing dataset size, Sachem/Lucy behaved more efficiently. The query processing time in Sachem/Lucy scaled sub-linearly with dataset size, showing only around $2\times$ slowdown on a $10\times$ larger dataset on median queries.

**Fig. 4:**
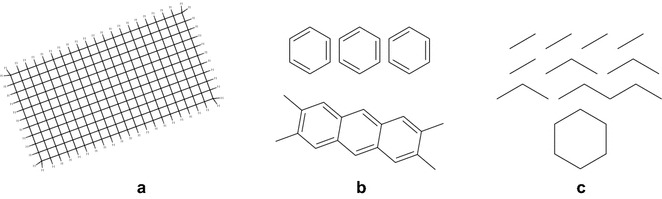
Noteworthy samples from three main classes of performance outliers identified during development: **a** PubChem compound CID20652954 (together with many similar compounds) is likely not a real molecule. Nevertheless, since there is no general method to identify a non-existent compound, it is not possible to reliably filter them out from the database. Trying to find an odd-length carbon cycle substructure in CID20652954 causes a complexity explosion; for example, matching the cycloheptadecane structure in it takes tens of minutes in all available cartridges before failing. **b** Matching a query that contains *n* benzene rings (above) in a compound that contains *n* or more benzene rings, but can only accommodate $n-1$ non-overlapping benzene rings (below) backtracks 12 times for each possible individual benzene position in the target molecule. In total, $\mathcal {O}((12n)^n)$ different atom permutations must be examined before the query fails. **c** A multi-fragment query that is too simple to produce any fingerprint information with enough filtering power for efficient screening. The performance of evaluating such queries mainly depends on the efficiency of data serialization and deserialization at the software interfaces between the back-end database and user

#### Performance outliers

Despite the performance improvements in the cartridges, there are several types of slow queries that are unlikely to be made more efficient by further development. Examples from the three main classes of such outliers are displayed in Fig. 4:

- Matching long cycles in dense structure graphs may cause an unavoidable complexity explosion, which is a problem inherent to graph substructure matching. Several examples of such dense graphs appear in the datasets.
- Matching multi-fragment queries in which each fragment may fit into multiple positions in the target causes a backtracking explosion, which takes $\mathcal {O}(m^n)$ time, where *n* is the number of fragments and *m* is the number of different embeddings of each fragment in the target.
- Screening can not reach the desired efficiency on queries that do not contain substructures with enough filtering power. These queries cause the cartridges to fetch large amounts of data from storage and run the verification algorithm on each of many identified positives.

### Screening efficiency

We compared the efficiencies of fingerprint-based screening processes in all cartridges in terms of precision (defined as the ratio of true positives to all identified positives identified by screening) and false positive rate (FPR, also known as fallout, defined as the ratio of false positives identified by screening to all negatives). Note that because the results from all cartridges differ slightly due to factors such as different perception of aromaticity and charges, the exact counts of positives and negatives are specific to each cartridge.

**Fig. 5:**
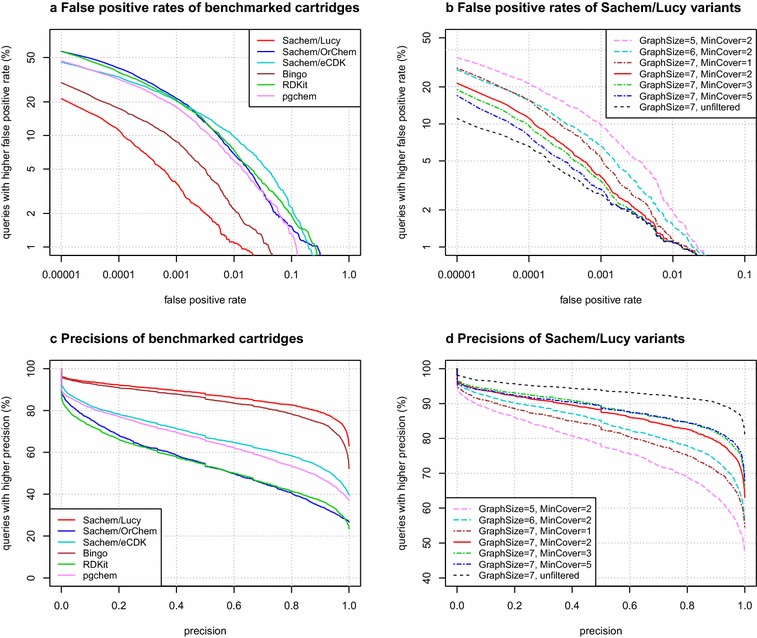
Screening efficiencies of different cartridges plotted as false positive rate and precision. OrChem values are not plotted because they are the same as those of Sachem/OrChem, which uses the same fingerprint. **a** Comparison of screening FPR for all queries in all cartridges; lower is better. Plotted values are the percent of queries from the query set that have a higher FPR when executed in given cartridge. For example, in Sachem/Lucy only $5\%$ of the queries have FPR worse than $10^{-3}$, and just under $80\%$ of the queries have negligible FPR (less than $10^{-5}$). **b** Same comparison for different parameters of Sachem/Lucy. The solid red line indicates the default parameters of Sachem/Lucy. **c** Comparison of the screening precision of all cartridges; higher is better. Plotted values are the percent of queries in the corresponding cartridge that have at least the given precision. **d** Same comparison for different variants of Sachem/Lucy

Plots for FPR and precision of all measured cartridges, displayed in Fig. 5, show the advantage of the screening method used in Sachem/Lucy over all other cartridges. Sachem/Lucy is followed by Bingo in terms of both precision and FPR. The remaining cartridges perform similarly, except for minor advantages of Sachem/eCDK and pgchem in precision.

#### Parameters of Sachem/Lucy

**Fig. 6:**
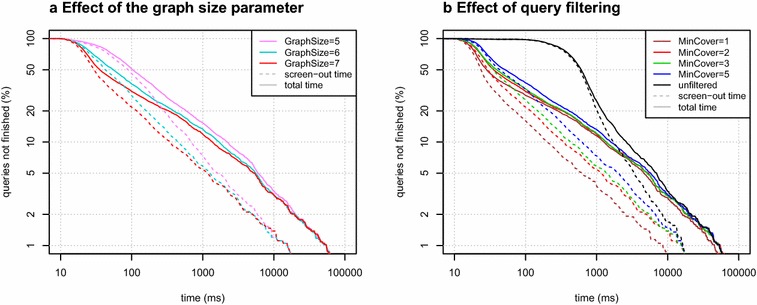
Effect of different choices of GraphSize and MinCover parameters on screening performance in Sachem/Lucy. **a** Increasing the fingerprint parameter GraphSize produces a larger fingerprint and larger indices, but overall performance is improved, thanks to the ability to choose a better set of fingerprints for screening. Note the log-time is plotted. **b** The same plot illustrating different choices of the MinCover parameter for the query filtering parameter. Unfiltered queries lose performance due to the overhead of storage access. Query filtering reduces this overhead while increasing the rate of false positives, thus increasing verification time. While MinCover = 1 might seem optimal, a larger value is beneficial for processing more complicated queries

We focused specifically on how varying the Sachem/Lucy parameters affects screening efficiency.

Reducing the GraphSize fingerprint parameter caused an expected increase in false positives, which projected to an increased query processing time (Fig. 6a). The default value GraphSize = 7 allows the implementation to pick fewer, more precise fingerprint bits. Specific deployments of Sachem might benefit from lowering the the graph size parameter to save storage space.

Query filtering had a considerable effect on query processing time. Although it caused a minor increase in false positives (as seen in Fig. 5b, d), it increased overall query performance. Because the processing time of unfiltered queries is, in most cases, clearly dominated by screening time, trading off some screening precision is beneficial. Setting the MinCover parameter to values as low as 1 can induce $10\times$ median speedup over unfiltered queries (see Fig. 6b). Screening time increases when the parameter is increased, but the overall query time does not seem seriously affected for values MinCover ≤ 5.

Although setting MinCover = 1 might seem optimal based on the results from the used query set, we use MinCover = 2 in Sachem/Lucy by default. This is substantiated by benchmarks on query sets that include larger queries, where the tradeoff is balanced differently. For example, finding a random subset of PubChem compounds in the 94M dataset runs optimally with parameter MinCover = 3, which is closely followed by MinCover = 2 (roughly 4% slower) and MinCover = 4 and 5 (9% and 15% slower). Setting MinCover = 1 is, in this case, almost 25% slower.

### Possible extensions and future goals

Sachem could easily be extended to similarity searches. The used text-search databases already recognize several notions of similarity measures, thresholds and top-N queries; after plugging in a matching similarity fingerprint, this functionality could be easily applied to high-performance similarity queries.

The ability to store and efficiently query fingerprints with a large number of keys is beneficial for several applications. For example, possible complications of substructure searches that arise from tautomerism or different perceptions of aromaticity could be resolved without significant impact on search performance. Simply indexing all possible tautomers or aromaticity variants is possible, at the cost of some storage space. An upper bound on the additional storage requirements can be estimated from the results of Sitzmann et al. [37].

Similarly, many non-structural and quantitative measures of molecules can be encoded to bit fingerprints, which can greatly simplify the processing of queries with heterogeneous parameters (sometimes called hybrid queries [38, section 2.4]). Consider a realistic query in which a researcher asks for a slightly alkaline compound with several substructures, available results in bioassays with high activity, limited molecular weight, and known binding with a protein. As this information is available in public databases, it could be easily converted to bit fingerprints and aggregated in a modified version of Sachem/Lucy. The resulting cartridge could answer any such query with similar performance as on the queries benchmarked in this paper. Alternative approaches to efficient querying of heterogeneous datasets in RDBMS include e. g. planner optimizations using cost estimation [39].

A simple adaptation to arbitrary fingerprints may also benefit drug discovery. Given the results of a bioassay, it is not known which feature of the molecule causes the desired activity, nor whether the activity is caused by anything relatable to an extant molecular fingerprint that could in turn be used to screen new candidates from molecule databases. Not being restricted by fingerprint size allows the researcher to easily define better fingerprints, in which individual bits may a have better chance to match the cause of the activity. Sachem can index compounds using even very large fingerprints, making such results of screening and analyses quickly available to the researcher.

Finally, the horizontal scaling potential of text-search databases like ElasticSearch or Solr [21, 22] could be easily exploited to provide a Google-like experience on a full-scale chemical substructure and similarity search. This is further supported by the fact that the software already supports Top-N queries and optimizations that are usually required to efficiently handle internet traffic.

## Conclusions

We have introduced Sachem, a new open-source cheminformatic cartridge oriented toward substructure search that improves the performance and scalability of substructural query processing.

Improvements in the OrChem-based indexing method enable Sachem/OrChem to process queries more than 50-fold faster than the original OrChem implementation. However, our results indicate that the original OrChem fingerprint design is still a viable choice for substructure screening.

The Sachem/Lucy variant, which is based on inverted indices, scales to very large datasets with similar or better performance than Sachem/OrChem on most dataset sizes. Compared to OrChem and other benchmarked methods, the Sachem/Lucy approach improves the precision of fingerprint-based screening. This variant stores a large fingerprint that identifies more than $10^7$ distinct features. The ability to store and efficiently query fingerprints of this size may benefit potential future applications of this method to more complicated datasets.

Both Sachem variants were benchmarked against other currently available open-source cartridges, using the PubChem database as a dataset and the SQC query set as queries. Sachem variants clearly outperformed other cartridges on most queries. Moreover, the performance of Sachem/Lucy was less affected by dataset size and fingerprint complexity, which is a required property to efficiently handle extremely large compound databases.

We expect that improvements in the performance and applicable size of screening fingerprints will simplify the deployment of substructure searches in new contexts, especially in prediction systems and heterogeneous databases .

## Availability and requirements

**Project name**: sachem

**Project home page**: http://bioinfo.uochb.cas.cz/sachem/

**Operating system(s)**: Linux

**Programming language**: C, C++, Java, SQL

**Other requirements**: Java 8 or higher, PostgreSQL 9.6 or higher.

**License**: GNU GPLv2

**Any restrictions to use by non-academics**: none other than those specified by the license.

## Footnotes

1 The growing demand for cartridge capacity is illustrated by the molecule count in PubChem, which has been doubling in size every 3 years. The growth is not expected to halt.

2 Radial and ECFP-like fingerprints [17] implicitly encode atom valences and thus are not generally applicable for substructure screening; a minor modification allows them to be used for queries in which the exact valence of atoms is requested.

3 For comparison, Webster’s Third New International Dictionary reports around a half million keywords in English language alone.
